# Pilot-scale conversion of lime-treated wheat straw into bioethanol: quality assessment of bioethanol and valorization of side streams by anaerobic digestion and combustion

**DOI:** 10.1186/1754-6834-1-14

**Published:** 2008-08-12

**Authors:** Ronald HW Maas, Robert R Bakker, Arjen R Boersma, Iemke Bisschops, Jan R Pels, Ed de Jong, Ruud A Weusthuis, Hans Reith

**Affiliations:** 1 Agrotechnology and Food Sciences Group, Wageningen University and Research Centre, PO Box 17, 6700 AA Wageningen, The Netherlands; 2 Energy Research Centre of The Netherlands, Biomass, Coal and Environmental Research, PO Box 1, 1755 ZG Petten, The Netherlands; 3 Lettinga Associates Foundation, PO Box 500, 6700 AM Wageningen, The Netherlands; 4 Nobilon Bacteriological R&D, P.O. Box 320, 5830 AH Boxmeer, The Netherlands; 5 Avantium Technologies BV, Zekeringstraat, 1014 BV Amsterdam, The Netherlands

## Abstract

**Introduction:**

The limited availability of fossil fuel sources, worldwide rising energy demands and anticipated climate changes attributed to an increase of greenhouse gasses are important driving forces for finding alternative energy sources. One approach to meeting the increasing energy demands and reduction of greenhouse gas emissions is by large-scale substitution of petrochemically derived transport fuels by the use of carbon dioxide-neutral biofuels, such as ethanol derived from lignocellulosic material.

**Results:**

This paper describes an integrated pilot-scale process where lime-treated wheat straw with a high dry-matter content (around 35% by weight) is converted to ethanol via simultaneous saccharification and fermentation by commercial hydrolytic enzymes and bakers' yeast (*Saccharomyces cerevisiae*). After 53 hours of incubation, an ethanol concentration of 21.4 g/liter was detected, corresponding to a 48% glucan-to-ethanol conversion of the theoretical maximum. The xylan fraction remained mostly in the soluble oligomeric form (52%) in the fermentation broth, probably due to the inability of this yeast to convert pentoses. A preliminary assessment of the distilled ethanol quality showed that it meets transportation ethanol fuel specifications. The distillation residue, which contained non-hydrolysable and non-fermentable (in)organic compounds, was divided into a liquid and solid fraction. The liquid fraction served as substrate for the production of biogas (methane), whereas the solid fraction functioned as fuel for thermal conversion (combustion), yielding thermal energy, which can be used for heat and power generation.

**Conclusion:**

Based on the achieved experimental values, 16.7 kg of pretreated wheat straw could be converted to 1.7 kg of ethanol, 1.1 kg of methane, 4.1 kg of carbon dioxide, around 3.4 kg of compost and 6.6 kg of lignin-rich residue. The higher heating value of the lignin-rich residue was 13.4 MJ thermal energy per kilogram (dry basis).

## Introduction

The limited availability of oil reserves and growing worldwide energy demands have resulted in increasing energy prices. Furthermore, the utilization of fossil fuels has negative impacts such as air pollution and the generation of the greenhouse gas carbon dioxide (CO_2_), which is presumed to be one of the main anthropogenic contributors to the global warming effect. These factors stimulate the exploitation of alternative renewable energy sources such as biomass [1]. According to the Kyoto protocols, many of the industrialized nations need to reduce their CO_2 _emissions by around 5% by 2010 as compared with the 1990 level, while a further decrease will be compulsory in the long term [2]. One strategy to meet the global increasing energy demand and the reduction of CO_2 _levels is the substitution of petrochemically derived transport fuels by CO_2_-neutral biofuels such as ethanol [3].

Bioethanol can be obtained by the microbial conversion of carbohydrates derived from biomass feedstocks such as agro-industrial residues [4-7]. Lignocellulose containing feedstocks are widely available, relatively inexpensive, non-competitive with food applications, sustainable in terms of CO_2 _emissions and, therefore, of potential interest for the large-scale production of bioethanol. Lignocellulose consists of a complex fibrous structure of polymeric sugars such as (hemi-)cellulose embedded in a matrix of the aromatic polymer lignin [4,8]. Wheat straw has been studied often as a raw material for microbial ethanol production processes [9-11]. The conventional lignocellulose-to-ethanol conversion consists of: (1) a pretreatment step; (2) a hydrolysis step; and (3) a fermentation step. Various physical and chemical pretreatments have been developed to alter the structure of lignocellulosic substrates [8]. An example is lime (Ca(OH)_2_) at mild temperatures (lower than 100°C), which enhances the accessibility of (hemi-)cellulose for the subsequent enzymatic hydrolysis [12-14]. We selected lime pretreatment as a model for further study as this has gained industrial interest because of its perceived advantages over other pretreatment methods, including the use of a low-cost chemical (lime) that is already in use in many agriculture-based, crop processing schemes (for example, sugar refining), lower reactor investment costs, as well as limited potential for the formation of degradation products. The hydrolysis, which is often performed by a mixture of cellulolytic and hemi-cellulolytic activities, results in a hydrolysate containing mainly soluble monosaccharides. Throughout the subsequent fermentation, these monomeric sugars can be converted into ethanol by yeasts or bacteria with a high productivity and efficiency. Alternatively, enzymatic saccharification and fermentation are performed simultaneously at compromising reaction conditions in one reactor. So far only a limited number of reports have been presented on the effects of lime pretreatment on the simultaneous saccharification and fermentation (SSF) of lignocellulosic biomass, including ethanol yield and the quality and valorization of side streams. Chang et al [15] submitted lime-pretreated switchgrass, corn stover and willow wood to laboratory-scale SSF and concluded that cellulose-derived glucose was extensively used by yeast to form ethanol during SSF, and that the pretreatment did not result in a significant inhibition of fermentation. In a more recent study, we investigated the use of lime-pretreated wheat straw for simultaneous hydrolysis and fermentation to lactic acid by an enzyme preparation and *Bacillus coagulans *DSM 2314 [16].

In a related study on the conversion of washed lime-treated wheat straw (LTWS) into ethanol, the optimal reaction conditions were determined on a laboratory scale [14]. The objective of the present paper is to study the effect of up-scaling on the performance of the lignocellulose-to-ethanol SSF process, quality assessment of the bioethanol and valorization of the by-product streams.

In the present study, ethanol was recovered from the fermentation broth via a distillation process and its composition was analyzed to assess its suitability as a transport fuel. The remaining distillation residue, consisting of water, lignin, non-hydrolyzed and non-fermented organic components, minerals from the feedstock, the added process chemicals and other non-ethanol fermentation products, was separated into a solid and liquid fraction. The combustion behavior of the solid fraction, which represents a significant part of the energy input, was studied and tested using a bench-scale experimental fluidized bed combustor (FBC) to assess the suitability of the solid fraction as fuel for combined heat and power generation. The liquid fraction, containing soluble organic components, was tested for its suitability for anaerobic biodegradation yielding biogas (methane). Utilization options for the ashes derived from combustion and sludge derived from anaerobic digestion are briefly discussed. This paper represents, to the best of the authors' knowledge, the first integral assessment of the conversion of lignocellulosic material into bioethanol including the valorization of side streams.

## Results

### Conversion of LTWS into ethanol by SSF

**Figure 1:**
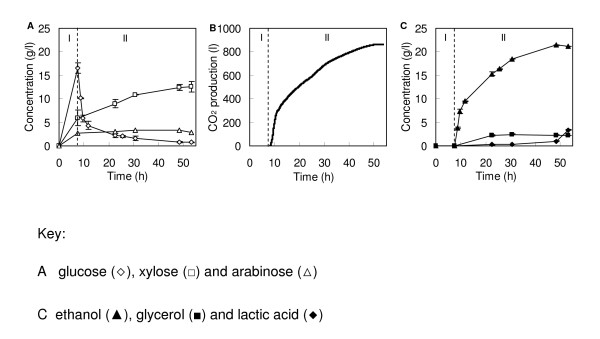
**Profiles throughout the simultaneous saccharification and fermentation of lime-treated wheat straw by commercial enzyme preparation and *Saccharomyces cerevisiae *at pH 5.0 and 37°C**. (A) The formation of sugars. (B) The production of carbon dioxide. (C) The production of fermentation products. The dotted line represents the addition of yeast and discriminates between a prehydrolysis phase (I) and simultaneous saccharification and fermentation phase (II). The error bars in the symbols denote the deviation of duplicate measurements.

After chemical treatment, the LTWS suspension was washed and dewatered as described previously [14]. The pretreatment was performed without significant loss of valuable fermentable sugars. The resulting biomass had a dry-matter content of approximately 35% by weight and contained 0.1 g/liter acetic acid. The polysaccharide composition of the washed/dewatered LTWS was (by weight) glucan 37.2%, xylan 20.1%, arabinan 2.8%, galactan 0.4%, rhamnan 0.2% and mannan 0.2%, whereas the remaining mass was primarily lignin 21.4%, uronic acids 1.6% and ash 12.6%. The polysaccharide fraction, glucan and xylan, accounted for 94% of the total polymeric sugars present in the LTWS. Therefore, we focused mainly on the conversion of these polysaccharides into fermentable sugars and ethanol via SSF in a 100-liter fermenter. The SSF process consisted of two phases, a prehydrolysis phase and an SSF phase (Figure 1), both controlled at 37°C and pH 5.0. The prehydrolysis phase aimed to reduce the viscosity (liquefaction) by adding LTWS manually together with the commercial enzyme preparation GC 220 containing hydrolytic enzymes. A total of 48.4 kg LTWS (16.7 kg dry matter) was added to the reactor during 6 hours of the prehydrolysis phase. Another 2 hours of prehydrolysis (total of 8 hours) resulted in a glucose, xylose and arabinose concentration of 15.4, 4.2 and 2.5 g/liter, respectively (Figure 1A). As soon as the bakers' yeast (*Saccharomyces cerevisiae*) was added to the reactor, the accumulated glucose was rapidly fermented to ethanol and carbon dioxide (Figure 1B and 1C).

The wild-type bakers' yeast only converts glucose and the pentose sugars, xylose and arabinose remain present in significant amounts. After 24 hours of incubation, the glucose concentration was low (less than 3 g/liter), suggesting that the enzymatic hydrolysis was the rate-limiting step. After 48 hours of incubation, the ethanol concentration increased to 21.4 g/liter corresponding to 48% of the theoretical maximum glucan-to-ethanol conversion. Based on this concentration, we concluded that 71% of the glucose monomers, liberated by hydrolysis, were converted into ethanol. This value is close to ± 80%, which is the theoretical maximum conversion of glucose by yeast under anaerobic growing conditions [17]. At the end of the SSF, a sudden production of lactic acid was observed, most probably as a result of microbial contamination by, for example, lactic acid bacteria (Figure 1C). During the SSF phase, the concentration of xylose increased to 11.8 g/liter, whereas the arabinose concentration remained constant at 3.3 g/liter (Figure 1A). The efficiency of the enzymatic saccharification and fermentation and the fate of the polymeric glucan and xylan after 53 hours of incubation are presented in Table 1. Part of the insoluble glucan initially present in the LTWS was still present as polysaccharide (25%), whereas minor amounts were present as oligomeric (6%) and monomeric sugars (1%). The largest part of the glucan was hydrolyzed and converted via glucose into fermentation products, such as ethanol (and CO_2_) (48%) and glycerol (5%), whereas the remaining part (15%) was most likely used mainly for yeast growth. The situation was different with xylan, which remained present as polysaccharide (26%), oligomeric sugars (52%) and xylose (24%).

**Table 1:** Fate of the polysaccharide glucan and xylan initially present in washed lime-treated wheat straw after 53 hours of simultaneous saccharification and fermentation

	**Fraction**	**Glucan (% by weight)**	**Xylan^c ^(% by weight)**
Hydrolysis products	Polysaccharides (insoluble)^a^	25	26
	Oligosaccharides (soluble)	6	52
	Monosaccharides (soluble)	1	24
Fermentation products	Ethanol and carbon dioxide (soluble)	48	---
	Glycerol (soluble)	5	---
Unaccounted for^b^	(Insoluble/soluble)	15	---

^a^A portion of the initial polysaccharides remained as insoluble polysaccharides.

^b^A portion of the initial polysaccharides were not recovered.

^c^Xylan-derived sugars were not converted by yeast.

### Quality of ethanol

To determine the quality of the produced ethanol for application as a transportation fuel, ethanol was distilled from the fermentation broth. The distillate contained an ethanol concentration of 11.5% by weight. The ethanol content was upgraded to approximately 90% by weight in a second distillation step. The distillate was subsequently analyzed for potential contaminants (Table 2). These results showed that the obtained product was fairly clean (on a water-free water basis the contaminant levels only slightly increase). With regard to the specifications for ethanol fuel described by the Detroit Diesel Corporation [18], the concentration of methanol, chloride ions and acetic acid were below the maximum limits. Furthermore, higher alcohols, esters or furfural type of components were not traceable in the ethanol distillate.

**Table 2:** Analysis of the ethanol distillate and comparison with ethanol fuel specifications [18]

**Component**	**Ethanol by fermentation**		**Specifications^a^**	
	**Ethanol distillate**	**Test method used**	**Values**	**Test method**

Ethanol	90.7% by weight (= 72.6% by volume)	Gas liquid chromatography using a flame ionization detector (GLC-FID)/Gas liquid chromatography-mass spectrometry (GLC-MS)	92% by volume (minimum)	ASTM D 3545-90
Methanol	0.01% by weight	GLC-FID/GLC-MS	2% by weight (max)	ASTM D 4815-89^c^
Anion content (for example, chloride)	Less than 1 mg/liter	Ion exchange chromatography	Chloride 0.0004% weight to weight (maximum)	ASTM D 3120-87^d^
Water	9.6% by weight	ASTM D 1364	0.5% by weight (maximum)	ASTM E 203-75
Acidity	0.0006% by weight HAc 0.01 mg potassium hydroxide per gram	ASTM D 1613	HAc 0.007% by weight (maximum) potassium hydroxide^b^	ASTM D 1613-85

^a^Specifications for E-95 (denatured) ethanol fuel according to the Detroit Diesel Corporation.

^b^No specifications available.

^c^Other alcohols and ethers.

^d^ASTM D 3120-87 modified for determination of organic chlorides and ASTM D 2988-86.

### Anaerobic conversion of the liquid fraction to biogas

**Figure 2:**
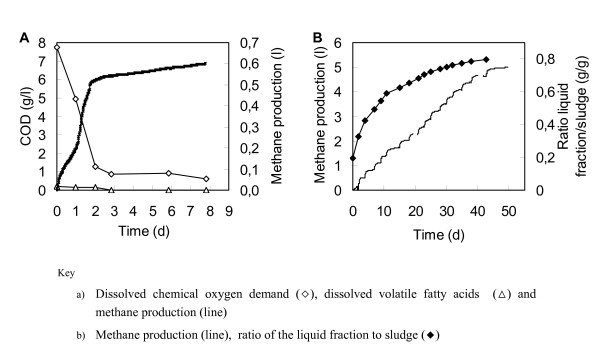
**Changes in dissolved chemical oxygen demand, volatile fatty acids (VFAs) and methane production during the anaerobic fermentation, and methane production during the accumulation test**. (A) Dissolved chemical oxygen demand, dissolved volatile fatty acids and methane production during the anaerobic biodegradability test with a ten times diluted liquid fraction (20 ml in a total of 200 ml). Data were corrected for the blank. (B) Methane production during the accumulation test where the ratio of the liquid fraction to sludge was increased. All data are represented as averages of duplicate experiments.

Directly after SSF and subsequent distillation, the distillation residue was collected and a solid/liquid separation was performed by centrifugation. Using this method, 71.6 kg distillation residue (17.3% dry matter and 9.7% insoluble solids) was divided into two fractions: 52.7 kg (10.9% dry matter) liquid fraction serving as a substrate for biogas production, and 18.6 kg (35.6% dry matter) of wet solid fraction functioning as a fuel for thermal conversion tests. The centrifugation step, however, was not optimized resulting in a liquid fraction containing a significant amount of solids present in the form of small particles such as fines and salts. The liquid fraction, including dissolved organic compounds (for example, xylose, arabinose, glycerol, acetic acid and lactic acid), was tested for potential anaerobic biodegradability to biogas (methane). Figure 2A shows the changes in dissolved chemical oxygen demand (COD), volatile fatty acids (VFAs) and methane production during the anaerobic fermentation. Throughout the fermentation, no VFA accumulation or acidification occurred (initial pH 6.7). The methane content of the biogas was 57% ± 5%. Furthermore, Table 3 shows an anaerobic biodegradability of 57% ± 1% on a COD basis. The remaining non-degraded fraction is most likely partly composed of non-biodegradable dissolved COD (Figure 2A) and partly of non- or very slowly biodegradable solid matter, as visual inspection after 8 days showed that the solid material present in the effluent had not disappeared. Table 3 shows a COD balance of the experiment. It can be concluded from the values that the dissolved fraction was almost completely degraded, whereas the non-dissolved organic matter was partially degraded.

**Table 3:** Chemical oxygen demand balance for the degradability of the liquid fraction (in grams of O_2 _based on a liter of effluent)

		**Chemical oxygen demand (g O_2_)**	**Percentage of initial total chemical oxygen demand value**
Initial state	Total	134.9	100
	Dissolved	54.6	40
	Non-dissolved^a^	80.3	60
Final state	Methane	77.2	57
	Dissolved	9.4	7
	Non-dissolved^a^	48.2	36

^a^Non-dissolved chemical oxygen demand was calculated from the measured values for total and dissolved chemical oxygen demand.

The effect of the potential presence of toxic components in the liquid fraction was tested by performing an accumulation experiment. Figure 2B shows the methane production in the accumulation test where the liquid fraction increased. No acidification took place and the average methane content of the biogas was 53% ± 2%. Roughly the same amount of methane was produced per feed, with an average degradability of 60% ± 1%. In conclusion, no adaptation of the sludge or toxic effects of the liquid fraction were observed suggesting feasible biodegradability of the liquid distillate residue.

### Thermal conversion of the solid fraction

The centrifuged wet solid fraction (with 27% moisture) was thermally dried (to 3.4% moisture) prior to laboratory-scale combustion tests. The composition of the solid fraction is important for its thermal conversion behavior. The chemical analysis of the solid fraction, together with the estimated composition corrected for 85% calcium recycle, original wheat straw and typical values for clean, untreated wood are presented in Table 4. The ash content of the experimental solid fraction was high (27.0% by weight) compared with straw (11.5% by weight) and wood (1.5% by weight). Furthermore, the silica content of the solid fraction increased significantly (by more than 4% by weight) compared with the wheat straw (2.8% by weight), due to the removal and conversion of cellulose and hemi-cellulose during upstream processes. As a result of the addition of calcium hydroxide and sulfuric acid during the pretreatment process and the absence of any calcium removal for recycling purposes, the concentrations of calcium (5.2% by weight) and sulfur (3.6% by weight) were increased compared with wheat straw and high compared with woody biomass fuels. If a calcium recycle is applied, these concentrations are estimated to be 0.8% by weight and 0.7% by weight, respectively. The content of phosphorus (0.51% by weight) increased compared with wheat straw owing to the addition of phosphorus containing additives during the fermentation process. Relatively small amounts of potassium (0.30% by weight) and chlorine (0.15% by weight) were found, more than in clean wood, but less than in the original straw as a result of the washing effect of water in the upstream processes. The lower heating value (LHV) of the LTWS solid fraction (experimental) was 12.4 MJ/kg (dry basis), which is lower in comparison with the original wheat straw feedstock (15.6 MJ/kg) and wood (18.5 MJ/kg), mainly because of the increased ash content. After mechanical dewatering of the solid fraction, a realistic LHV of the solid fraction is estimated at 5 MJ/kg (wet basis) assuming that 50% moisture in the fuel will remain for a process at industrial scale.

**Table 4:** Fuel analysis results of the lime-treated wheat straw solid fraction (experimental and corrected for 85% calcium recycle) original wheat straw feedstock and untreated wood

**Source composition**		**Lime-treated wheat straw solid fraction (experimental) **	**Lime-treated wheat straw solid fraction (corrected for 85% calcium recycle)**	**Wheat straw original feedstock (harvest 2003)**	**Wood (untreated, average) **[28]
Parameter	Unit				

Ash (550°C)	wt%_dry_	27.2		12.6	---
Ash (815°C)	wt%_dry_	27.0	19.5	11.5	1.5
Volatiles	wt%_dry_	68.4	75.5	70.1	80.7
Moisture	wt%_wet_	3.4^a^	50^b^	7.7	49.2
Higher heating value, measured	MJ/kg_dry_	13.4	14.3	16.6	19.8
Lower heating value	MJ/kg_dry_	12.4	13.2	15.6	18.5
Lower heating value (at 50% MC)	MJ/kg_wet_	5.0	5.4	6.6	8.0
					
C	wt%_daf_	43.1	44.9	46.5	51
H	wt%_daf_	5.8	6.1	5.6	6.1
N	wt%_daf_	1.5	1.5	1.7	0.3
O	wt%_daf_	44.8	46.6	45.8	43
S	wt%_daf_	4.56	0.71	0.15	0.06
Cl	wt%_daf_	0.20	0.21	0.31	0.04
F	wt%_daf_	0.0031	0.0031	---	0.0024
Al	mg/kg_dry_	121	133	185	211
As	mg/kg_dry_	5.4	5.9	< dl	less than dl
B	mg/kg_dry_	5.2	5.7	4.1	9
Ba	mg/kg_dry_	3.2	3.5	3.6	143
Ca	mg/kg_dry_	52000	8600	4700	13000
Cd	mg/kg_dry_	Less than dl	---	0.15	0.5
Co	mg/kg_dry_	7.3	8.1	55	0.51
Cr	mg/kg_dry_	10.2	11.2	0.2	5
Cu	mg/kg_dry_	9.1	10.1	1.6	17
Fe	mg/kg_dry_	160	170	220	120
K	mg/kg_dry_	3000	3300	15000	2000
Li	mg/kg_dry_	Less than dl	---	0.2	Less than dl
Mg	mg/kg_dry_	870	36	1105	769
Mn	mg/kg_dry_	33	36	14	214
Mo	mg/kg_dry_	Less than dl	---	1.9	0.55
Na	mg/kg_dry_	1500	1660	200	300
Ni	mg/kg_dry_	5	5.8	0	8
P	mg/kg_dry_	5100	5600	485	486
Pb	mg/kg_dry_	Less than dl	---	1.1	32
Sb	mg/kg_dry_	1.4	1.6	1.8	1.8
Se	mg/kg_dry_	Less than dl	---	1.9	1
Si	mg/kg_dry_	40000	44500	28000	900
Sn	mg/kg_dry_	0	0	0.20	1.2
Sr	mg/kg_dry_	23	25	18	14
V	mg/kg_dry_	0.3	0.3	---	---
Zn	mg/kg_dry_	25	27	0.6	0.4

^a^After thermal drying.

^b^After mechanical dewatering with Pneumapress.

No single agglomeration index has been developed that reliably describes the agglomeration behavior of a biomass fuel under all combustion conditions [19]. The agglomeration indicators presented in Table 5 only give a rough indication and should be interpreted with care. The indicators suggest a tendency for smelt-induced agglomeration for the fuels. The indicators for fouling suggest that there is less risk of fouling.

**Table 5:** Agglomeration and fouling indicators of the lime-treated wheat straw solid fraction (experimental and corrected for 85% calcium recycle) original wheat straw feedstock and untreated wood

**Source composition**		**Lime-treated wheat straw solid fraction (experimental)**	**Lime-treated wheat straw solid fraction (corrected for 85% calcium recycle)**	**Wheat straw original feedstock (harvest 2003)**	**Wood (untreated, average) **[28]
**Fluidized bed combustion agglomeration indicators^a^**					
*Sinter-induced*^*b*^					
(1) Na + K/2S + Cl more than 1		0.1	0.4	2.7	1.4
(2) (Na + K + Si)/(Ca + P + Mg) more than 2		0.8	3	7.0	0.2
					
*Smelt-induced*^*c*^					
(3a) K + Na/Si more than 0.6		0.1	0.1	0.5	2.6
(3b) K + Na more than 1 g/kg		5	5	15	2.3
(3c) Si more than 1.5 g/kg		40	44	28	0.9
(3d) K_2_O + Na_2_O + SiO_2 _more than 50% ash		34	52	68	30
					
**Fluidized bed combustion fouling indicators (at 50% moisture content)^a^**					
3 Cl +S more than 1% weight_ar_		2.1	0.6	0.9	0.2
Na + K + Zn more than 1% weight_ar_		0.2	0.3	0.4	0.2
Dust fraction of ash more than 30%		Unknown	Unknown		Unknown
Alkali/DOE index^d^	kg/GJ HHV^e^	0.3	---	0.9	0.1

^a^Visser and Laughlin [29].

^b^The second indicator is relevant if the first indicator is exceeded.

^c^The more the indicators exceed the limit value, the more likely it is that smelt bed agglomeration will occur.

^d^Miles et al [30]: if the value of the index is higher than 0.17, probably fouling; if more than 0.34, certainly fouling.

^e^Higher heating value.

During the combustion test, no signs of bed agglomeration were observed. As the experiment lasted for only 4 hours, care should be taken to extrapolate this observation to the long-term combustion behavior of the fuels. Longer tests have to be performed for a more profound assessment. A scanning electron microscope was used to identify the possible build-up of a coating layer on the bed material (sand), which can indicate possible agglomeration. No potassium silica coating could be detected on the bed material after the experiments with ethanol fermentation residues.

Owing to the higher sulfur content, sulfur dioxide (SO_2_) emissions will be a point of attention and are significantly different from the regular combustion of wood, but well-known in coal and straw-fired combustion installations. The measured hydrogen chloride (HCl) concentration in the flue gas during the lime pre-treated residue combustion was 180/190 mg/Nm^3 ^(at 6% volume O_2_) and the SO_2 _concentration was 3000/3360 mg/Nm^3 ^(at 6% volume O_2_). The SO_2 _concentrations are high as a result of the absence of a calcium recycle during the upstream fermentation process in the pilot-scale fermentation tests. This calcium recycle is, however, foreseen at the industrial scale. A large fraction of sulfur is bound to the calcium present in the solid residue as gypsum and, therefore, not released into the flue gas. With a calcium recycle, the SO_2 _flue gas concentration is expected to be 85% lower, assuming the same ratio between sulfur and calcium (resulting in the same sulfur capture ratio).

A deposition probe was placed in the flue gas for the duration of the whole test (4 hours). After finishing the experiment, the probe was carefully removed and visually inspected. Compared with wood combustion, the deposition rates of ash particles on the probe were high due to the much higher ash content of the fermentation residues. The ash depositions could be easily removed from the surface and appeared to be non-sticky. A facility for deposition removal to keep the boiler tubes as clean as possible, such as a knocking mechanism, is therefore recommended on an industrial scale, as is commonly used in, for example, pulverized coal-fired power plants. No further analyses on the probe have been performed, as no significant effects on the probe material are expected due to the limited duration of the experiments.

Combustion in a circulating fluidized bed (CFB) was selected as the most suitable conversion technology due to the small particle size of the lignin residue (less than a few millimeters), moisture content (approximately 50% to 60% after dewatering) and the thermal capacity. The combustion temperature in a CFB is relatively low (800 to 900°C) to reduce the risk of sintering of the ashes in the bed. To reduce the risk of agglomeration, it is possible to partially refresh the bed material during operation. It is expected that emission standards can be met in a full-scale process by standard flue gas cleaning technologies (for example, flue gas sulfurization, de-NO_*x*_, particle removal).

### Overall balance

**Figure 3:**
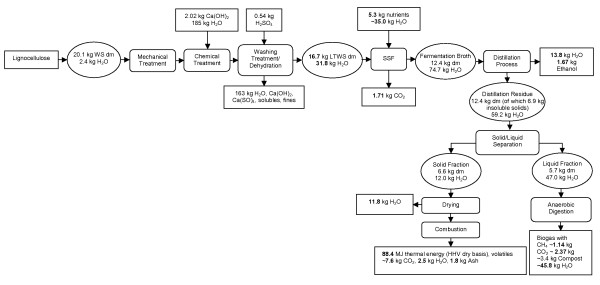
**Schematic depiction of an integrated process converting lignocellulose into ethanol, biogas (methane), carbon dioxide, compost, energy (heat and power) and ash**. The energy can be used for the process or delivered to the grid. The 'clean' water can be recycled into several steps of the process.

An overview of the lignocellulose-to-ethanol process with the respective process steps of side streams is presented in Figure 3. Furthermore, the input and output streams were quantified. With the results obtained by the SSF to ethanol, anaerobic digestion to methane and thermal conversion, we calculated that 1000 kg of LTWS can be converted into 100 kg of ethanol (equal to 3.0 GJ thermal energy calculated with a higher heating value (HHV) of 29.7 MJ/kg), 69 kg methane (equal to 3.8 GJ thermal energy calculated with an HHV of 55.5 MJ/kg), approximately 204 kg sludge, 246 kg CO_2 _and 398 kg lignin-rich residue. The solid lignin-rich fraction contains an HHV of 13.4 MJ/kg (dry basis) equal to a total of 5.3 GJ of thermal energy for heat and power generation. Furthermore, the combustion of the lignin-rich fraction results mainly in 458 kg CO_2_, 151 kg water and 107 kg inorganic ash.

## Discussion

Lignocellulosic biomass has great potential with regard to its use as a substrate for the large-scale production of biofuels. The lignocellulose-to-ethanol conversion via SSF is a complex process involving various enzymatic and microbial activities. Results from previous studies showed that the mild-temperature lime treatment (0.1 g Ca(OH)_2 _per gram substrate, 16 to 20 hours, at 85°C) enhances the accessibility of polymeric carbohydrates towards hydrolytic enzymes resulting in the release of fermentable sugars and improved ethanol yield [14]. Nevertheless, these studies were performed at the laboratory and bench scale. This paper describes the pretreatment, enzymatic hydrolysis and microbial conversion of LTWS via SSF into ethanol on a pilot scale. Prior to using the lime-treated substrate for hydrolysis and fermentation, the inhibitor acetic acid was successfully removed by a relatively simple washing procedure.

Reaction conditions and enzyme loadings determined in previous optimization studies were used for hydrolysis and fermentation. This resulted in a glucan-to-ethanol conversion (48%), which is lower than the yields obtained in laboratory tests (56%) [14]. An explanation for this difference in ethanol yield can be found in the higher dry-matter content, which was approximately twice as high in the pilot-scale ethanol fermentation (around 20% by weight), negatively influencing the enzymatic hydrolysis. A possible explanation for the low glucan-to-ethanol yield can be found in the fact that xylose is present in the medium and may inhibit the activity of the cellulolytic complex [20]. Another possible explanation for the lower yield is that in this study the lime pretreatment was carried out on a pilot scale and without continuous temperature control, thereby leading to lower cellulose digestibility compared with a bench-scale test. Approximately 25% of the glucan in the LTWS remained present as an insoluble polymeric sugar suggesting the presence of crystalline and hardly degradable cellulose and/or inactivation of the hydrolytic system. Our findings support the results of Chang et al [15] who reported that enzymatically hydrolyzed cellulose from lime-pretreated biomass was utilized extensively by yeast, suggesting that the fermentations were not limited by the glucose metabolism of the yeast.

The situation is different in the case of xylan. Owing to the inability of the wild-type baker's yeast to convert pentose sugars, end-product inhibition by xylose possibly occurs resulting in the accumulation of mainly xylo-oligomeric sugars. Previous SSF work with LTWS, hydrolytic enzymes and *Bacillus coagulans*, a pentose-converting, lactic-acid-producing bacterium, showed that the concentration of xylo-oligomeric sugars and xylose remain low [16]. This indicates that the presence of xylose monomers in the medium of the pilot-scale ethanol process possibly inhibit the hydrolysis of soluble xylo-oligomeric sugars. In addition, the xylan-to-xylose conversion at pilot-scale SSF (24%) agrees relatively well with the yield obtained in laboratory-scale SSF experiments (28%). Further optimization of the enzymatic hydrolysis and fermentation step can be realized by the use of pentose-converting yeast resulting in a higher ethanol concentration and yield.

If a xylose-converting yeast is used, an additional amount of ethanol approximately 1.4 kg (assuming the conversion of 74% of xylan, which is the fraction that was present as a monomer and oligomer after 53 hours of incubation, with an ethanol yield of 0.51 g/g) can be formed from this C_5 _sugar. Less dissolved substrate will be available for anaerobic digestion resulting in an approximately 55% reduction of methane to around 0.5 kg.

Ethanol was upgraded via two separate distillation processes resulting in a distillate containing 90% ethanol by weight. Tests showed that the ethanol distillate, in comparison with the ethanol fuel specifications of the Detroit Diesel Corporation, seems suitable as a transportation fuel.

The fermentation residue was separated into two fractions; one fraction contained mainly solids functioning as a fuel for thermal conversion and the other was a liquid fraction that contained mainly soluble compounds serving as a substrate for anaerobic fermentation to biogas. The liquid fraction had a moderate anaerobic biodegradability of 60% respective to the total COD content and no signs of toxicity could be observed in the accumulation test. The relatively low value is largely a result of the high amount of solid organic matter in the liquid fraction, which was only partly degraded. More efficient removal of the solid matter would contribute to a significant reduction of the COD load. As the dissolved matter has a high anaerobic biodegradability, anaerobic treatment would then be an attractive way of processing this effluent for COD reduction and energy production in the form of methane gas.

The solid fraction of the distillation residue appeared to be suitable as a fuel for thermal conversion, although it contained a relatively high ash concentration and relatively high concentrations of sulfur and calcium due to additions in the upstream processes. It is expected that the combustion will show a behavior between the original feedstock wheat straw and (clean) wood. Based on the analyzed residue composition, there is some risk of smelt-induced bed agglomeration. The risk of sinter-induced bed agglomeration is expected to be less than for straw, as a result of the partial removal of potassium (washing) in the upstream processes.

The combustion experiments showed no agglomeration and no extensive coating was detected on the bed material that would indicate agglomeration. Long-duration experiments on a larger scale are necessary, however, to provide a more profound assessment of the fuel behavior. With respect to fouling, high ash deposition rates were observed. The composition of both bottom ashes and fly ashes were determined and it is most likely that both could be utilized rather than being land-filled. The main options are utilization as building materials and, in some cases, as fertilizers. A more conclusive assessment of the potential utilization of the ashes requires larger samples that are more representative of the full scale, because the ashes from the pilot-scale test likely have a lower salt content. Details on the assessment of ashes will be presented in a follow-up paper.

## Conclusion

This paper describes the successful up-scaling of the conversion of high dry-matter content LTWS via SSF into bioethanol on a pilot scale. In comparison to conversions obtained in laboratory experiments, we concluded that after 48 hours of incubation, slightly lower fermentable sugar and ethanol yields were achieved at the pilot scale. Tests showed that the produced ethanol was suitable as a transportation fuel. The side streams were quantitatively analyzed. The liquid fraction containing mainly soluble components was valorized by anaerobic fermentation to biogas (methane) and sludge. The remaining solid fraction was assessed as a fuel for conversion by combustion yielding thermal energy and inorganic ashes with the prospective of being utilized rather than being land-filled.

## Methods

### Lignocellulosic feedstock, enzymes and yeast

Wheat straw was purchased from a farm located in the northeast of The Netherlands. The wheat straw was air-dried (89.5% dry matter by weight) and ground to pass through a 4-mm screen. The chemical composition of the wheat straw was determined by using a modified TAPPI Tcm-249 test method as described elsewhere [21]: glucan 33.3%; xylan 19.8%; arabinan 2.2%; rhamnan 0.3%; mannan 1.1%; lignin 21.8%; uronic acids 2.5%; and extractives 10.7%. The commercial enzyme cocktail GC 220 (Genencor-Danisco, Rochester, NY, US) contained cellulase (filter paper units), cellobiase and xylanase activities of 116, 215 and 677 U/ml, respectively [22], with a specific gravity of 1.2 g/ml. The ethanol fermentation was performed with the wild-type bakers' yeast (Koningsgist, DSM, The Netherlands). Chemicals, unless indicated otherwise, were purchased from Merck (Darmstadt, Germany).

### Alkaline pretreatment and washing procedure

The alkaline pretreatment of air-dried wheat straw was performed in a 215-liter heated vessel. About 22.5 kg ground wheat straw (approximately 20.1 liters per kilogram of dry matter) was moistened with 40 liters of water and placed in the vessel. Subsequently, 2019 g of lime was dissolved in 145 liters of water and added to the vessel and the resulting suspension was then heated to 80–85°C under continuous mixing by a stirrer. After 8 hours of incubation (80–85°C), the vessel was thermally isolated and throughout the following 16 hours of incubation, the temperature of the wheat straw suspension decreased to 66°C. After a total of 24 hours of incubation, the LTWS was transferred to a drainage vessel and dewatered through two sieves (pore sizes 3 mm and 0.3 mm, respectively) by gravitation. The solid fraction was returned, together with 215 liters of fresh hot water (55 to 60°C), into the drainage vessel, continuously mixed for 30 minutes, and dewatered using the same approach outlined above. The washing/dewatering procedure was repeated five times. The pH of the pulp was adjusted to pH 6.5 with a 25% (by volume) sulfuric acid (H_2_SO_4_) solution. Finally, the pulp was dewatered once more to a dry-matter content (105°C, 16 hours) of approximately 35% and stored at 4°C.

For the determination of the insoluble solid content, the samples were centrifuged for 5 minutes at 3000 rpm, supernatant was removed and the remaining pellet was washed by re-suspension with water. The sequence of washing and centrifugation was repeated three times followed by drying the pellet overnight at 105°C.

### Simultaneous saccharification and fermentation

The SSF of LTWS was carried out in a 100-liter fermenter (Applikon, Schiedam, The Netherlands) with a pH and temperature control (Biocontroller ADI 1020). The reactor was filled with 35 liters of tap water. The following salts were added to the water: ammonium sulfate ((NH_4_)_2_SO_4_), 425 g; monopotassium phosphate (KH_2_PO_4_), 255 g; and magnesium sulfate (Mg_2_SO_4_.7H_2_O), 42.5 g. The pH was adjusted and maintained at 5.0 with a 2 M H_2_SO_4 _solution and a 20% (weight to volume) Ca(OH)_2 _suspension. The process started with a prehydrolysis phase (I) of 8 hours at 37°C and an agitation rate varying between 450 rpm and 700 rpm. During this phase, 48.42 kg of washed LTWS (with a dry-matter content of approximately 35%) and 1105 ml GC 220 were added manually. The rate of substrate addition depended on the viscosity of the LTWS suspension in the reactor. After prehydrolysis, the following components were added to the LTWS: trace element solution, 85 ml; vitamin solution, 85 ml; fatty acid solution, 106.25 ml; anti-foam Acepol 77 (Emerald Foam Control), 42.5 ml; antibiotics penicillin and steptomycin (Sigma-Aldrich), 212.5 ml; second dosage of enzyme preparation GC 220, 787 ml; and fresh wet bakers' yeast (Koningsgist, DSM, The Netherlands), 1071 g (around 353 g dry yeast, 4.2 g dry yeast/liter fermentation broth), all according to the methods described previously [14]. The SSF phase (II) was initiated by the addition of yeast cells and the reactor was closed in order to analyze CO_2 _production (Uras 10E, Hartmann & Braunn). A nitrogen gas flow between 5 and 40 liters/minute was added to the fermenter functioning as a carrier gas of the produced CO_2_. As CO_2 _is produced in equimolar amounts with ethanol from glucose by bakers' yeast, measurements of CO_2 _production rates can be used to estimate the production rate of ethanol. Samples were taken throughout the experiment, treated and analyzed for the composition of monomeric sugars, organic acids, glycerol and ethanol by high-pressure liquid chromatography methods as described previously [14]. The soluble oligomeric sugars and insoluble polymeric sugars were determined as described previously [16].

### Recovery of ethanol and quality assessment

After completion of the SSF process, the fermentation broth in the fermenter was heated up to 83°C. Nitrogen gas (15 liters/minute) functioned as a carrier gas of evaporated ethanol. The ethanol was condensed by a cold fall spiral (length 270 cm, diameter 2 mm), which was cooled by water at 4°C. The distillate fraction of condensed ethanol/water mixture was collected in a bottle cooled with melting ice water.

Further upgrading of the ethanol content was performed in a second distillation step, which was carried out in a batch distillation unit with a 3-liter boiler volume. An Oldershaw-type distillation column containing 28 theoretical stages was used. The boiler was filled with 2 to 2.5 liters of the ethanol/water mixture obtained in the first distillation step. Hence, several distillation runs were required to produce sufficient material. Distillate fractions of approximately 20 g were collected separately and analyzed on ethanol content. During the first phase of the distillation, the reflux ratio was kept at 2. After receiving the first 100 g, the reflux ratio was increased to 3. This was done to ensure the required ethanol content of the distillate (during a batch distillation the most volatile component is continuously depleted from the liquid in the boiler). All distillate fractions were analyzed for their ethanol content by gas liquid chromatography using a flame ionization detector (GLC-FID). The final distillate sample (all combined distillate fractions with sufficient ethanol content) was analyzed for water content (Karl Fischer titration, ASTM D 1364), acidity (acid-base titration, ASTM D 1613), anion content (chloride, sulphate, nitrate and carbonate measured by ion exchange chromatography (ICE)) and organic components other than ethanol (gas liquid chromatography-mass spectrometry (GLC-MS)).

### Solid/liquid separation of distillation residue

After the distillation step, the distillation residue was separated by centrifugation (Heine Zentrifuge, type DM-K-Z 2237-2V2, 1977), resulting in a liquid fraction and a fraction containing mainly solid particles. The feeding rate of the distillation residue was around 6 liters/minute with an agitation rate of 3500 rpm.

### Anaerobic biodegradation of the liquid fraction and biogas production

The anaerobic biodegradability of the liquid fraction to methane was studied by adding 20 ml liquid fraction to 21.3 ml of macronutrients, trace elements and 10 mM potassium/sodium phosphate buffer (pH 7) [23], inoculated with 75 g wet sludge. The mixture was adjusted with water to 200 ml total volume and incubated at 30°C. The inoculum used was anaerobic granular sludge from a reactor treating potato-processing wastewater with an organic matter content of 0.08 g/g sludge. The sludge concentration was 30 g/liter. A blank without sludge was used to correct for methane production from the inoculum itself. During the test, gas production and composition, pH, COD and VFAs were measured. The dissolved COD concentration was determined by the micromethod with dichromate (Dr Lange, Germany). For determination of the VFA concentration, samples were withdrawn, centrifuged for 10 minutes at 10000 rpm and analyzed on a gas chromatograph (Hewlett Packard 5890 series II) with a 30 m Alltech column (AT-Aquawax-DA 0.32 mm × 0.25 mm). The temperatures of the column, injection port and FID were 80°C (beginning) to 220°C (end), 275°C and 300°C, respectively. Nitrogen saturated with formic acid was used as the carrier gas (20 ml/minute). The volume of produced biogas was determined using the Oxitop pressure measurement system. The quantities of these components in the reactor gas were determined on a gas chromatograph (Hewlett Packard 5890) with two different columns: 30 m Molsieve 5A 0.53 mm × 15 μm and 25 m Poraplot Q 0.53 mm × 20 μm. The carrier gas was helium and the temperatures of the column, injection port and the thermal conductivity detector were 45°C, 110°C and 99°C, respectively.

The potential toxicity of the liquid fraction was studied in an accumulation test with an undiluted liquid fraction. The liquid fraction was added to the reactors in small quantities, increasing the concentration with each addition until a final concentration of 80% liquid fraction was reached with respect to the substrate/sludge relation (owing to time limitations, reaching 100% was not possible). With each liquid fraction addition, macronutrients, trace elements and phosphate buffer were also added to the system. The test was carried out at 30°C. The initial sludge concentration was 63 g organic matter per liter, after all additions this was reduced to 15 g/liter. A blank without a liquid fraction was used to correct for methane production from the inoculum itself. During the test, gas production and composition were measured, as well as the pH, to check for acidification of the system.

### Determination of the quality of the solid fraction for combustion

The solid fraction was assessed as a fuel for combustion. It was analyzed using standard methods for the chemical analysis of biomass fuels according to the Dutch NEN NTA 8200 series. The results were used to calculate fluidized bed combustion agglomeration and fouling indicators. Suitable conversion technology was selected using in-house expertise and data from the literature [19,24-26]. Combustion experiments were performed in a bench-scale experimental FBC (feed rate approximately 0.15 kg/hour) [27]. The reactor had a diameter of 7 cm (bottom) and 11 cm (top) and a height of 110 cm and was made of 253 MA. Air was used to fluidize the sand bed. The FBC was operated at a temperature of approximately 850°C and 5% to 6% oxygen by volume in the dry flue gas. To prevent heat losses, the set-up was electrically externally heated by tracing elements. The solid fraction was thermally dried before combustion (approximately 3% moisture by weight) and milled (2 mm sieve diameter). The fuel was transported into the reactor by a screw feeder. During combustion of the fermentation residues, the fly ash was entrained with the flue gas and collected by Soxhlet filters (200°C). The flue gas was analyzed online for: NO_*x*_, O_2_, CO_2 _and SO_2_, and off-line (wet chemical) for SO_2 _and HCl. A deposition probe, cooled with water, was placed into the flue gas to simulate boiling tubes in a boiler. After finishing the experiment, the probe was carefully removed and visually inspected.

## Competing interests

The authors declare that they have no competing interests.

## List of abbreviations

CFB: circulating fluidized bed; COD: chemical oxygen demand; FBC: fluidized bed combustor; FID: flame ionization detector; GLC-FID: gas liquid chromatography using a flame ionization detector; GLC-MS: gas liquid chromatography-mass spectrometry; HHV: higher heating value; ICE: ion exchange chromatography; LHV: lower heating value; LTWS: lime-treated wheat straw; SSF: simultaneous saccharification and fermentation; VFA: volatile fatty acid.

## Authors' contributions

RHWM carried out the SSF experiment and drafted the manuscript. RRB performed the alkaline pretreatment and washing procedure and helped to draft the manuscript. ARB carried out the thermal conversion of the solid fraction via combustion and helped to draft the manuscript. IB carried out the anaerobic conversion of the liquid fraction and helped to draft the manuscript. JRP performed the ash quality analysis and helped to draft the manuscript. EJ participated in the design and coordination of the study and helped with the final discussion of the manuscript. RAW participated in the design and coordination of the study and helped to draft the manuscript. HR participated in the design and coordination of the study and helped with the final discussion of the manuscript.
